# Short-Spindled Cell Haemangioblastoma with CD34 Expression: New Histopathological Variant or Just a Stochastic Cytological Singularity?

**DOI:** 10.1155/2016/6749590

**Published:** 2016-05-25

**Authors:** Miguel Fdo. Salazar, Paola Andrea Escalante Abril, María Verónica Velasco Vales, Celene Martínez Ruiz, Erick Gómez Apo, Laura G. Chávez Macías

**Affiliations:** Pathology Unit, Neuropathology Service, Mexico General Hospital, 06726 Cuauhtémoc, DF, Mexico

## Abstract

Haemangioblastomas are neoplasms of uncertain histogenesis with cellular and reticular variants advocated in current lore. Herein we describe an intriguing cerebellar specimen with unusual traits including spindle cell morphology and CD34 positivity. A thirty-nine-year old man had an infratentorial tumour discovered incidentally and resected three times. In all the instances, histopathological diagnosis was haemangioblastoma; nonetheless, he had neither physical stigmata nor family history of von Hippel-Lindau disease. By histology, the lesion was composed of areas of conventional stromal cells admixed with territories populated by short-spindled cells packed in lobules, sometimes giving the appearance of* gomitoli*. Immunoperoxidase-coupled reactions confirmed the expression of inhibin A, neuron-specific enolase (NSE), PS100, and CD57 but also revealed focal immunolabeling for CD34, CD99, and FXIIIa. This case highlights the potential phenotypical diversity that can be found within these neoplasms. Rather than uncertain histogenesis, it may in fact reflect multiple lines of differentiation—*histomimesis*—prone to adopt unusual morpho- and immunophenotypes in a subset of haemangioblastomas.

## 1. Introduction

Haemangioblastomas are neoplasms of uncertain histogenesis comprising stromal cells amidst a rich capillary network with cellular and reticular variants advocated in current lore [1, 2]. Interestingly, there appear to be some instances in which the cell phenotype experiments changes that seem to vaguely pull them away from these two histological standards. Herein we document a case displaying an unusual spindle cell shape with expression of CD34.

## 2. Case Report

**Figure 1:**
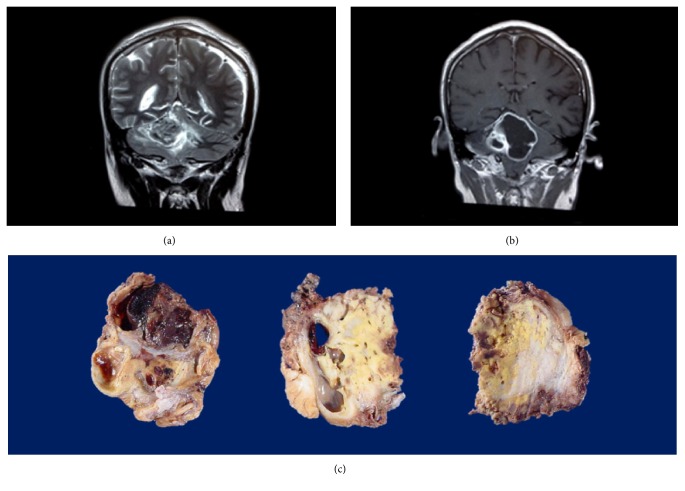
Magnetic Resonance Imaging Scans/Specimen Gross Findings. (a) T_2_-weighted coronal section at the time of admission. There is a predominantly solid lesion in the right cerebellar hemisphere. (b) Postcontrast T_1_-weighted image in coronal plane. A cystic component with peripheral enhancement has appeared. (c) Third specimen serial slices. Yellow-hued areas experiment sudden transition into gray-opaque zones, which microscopically corresponded to the spindled stromal cell population.

A 37-year-old man was brought to medical examination after suffering head trauma. Early imaging scans unexpectedly disclosed a right cerebellar mass of heterogeneous intensity compressing the fourth ventricle and without attachment to the* tentorium cerebelli* (Figure 1(a)). Despite tumour resection, new scans requested four months later showed persistence of the tumoural mass with roughly the same size. Again, removal was practiced. Regardless of both interventions, the lesion prevailed and had acquired a cystic appearance (Figure 1(b)). It was excised once more. The surgical piece received displayed the classic traits of haemangioblastoma—yellow spots and blood filled cysts—but also showed pale-gray, fibrous areas (Figure 1(c)). The patient was subsequently followed up to discard diagnosis of von Hippel-Lindau disease; three years later, however, no additional stigma has been found. He is also known to lack any family history related to this syndrome.

**Figure 2:**
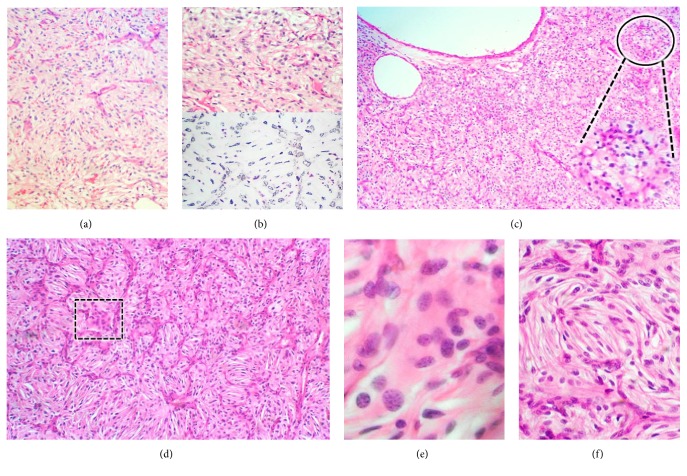
Light Microscopy Morphophenotype. (a) Spindle cells in the first resection sample. (b) Second resection sample: spindle cell component seen with haematoxylin and eosin (upper field) and with reticular fibers stain (lower field). The reticulin pattern is absent in the latter. (c) Third resection sample: transition area between fusiform stromal cells (left field) and conventional stromal cells (right field and lower inset). (d) Panoramic view of spindle cells in the third resection sample. A multinucleated giant cell can also be recognised (dotted-lined square). (e) High magnification photomicrograph of a syncytium-like cell. (f) Glomeruloid or yarn ball cluster of short-spindled cells “*gomitoli.*”

**Figure 3:**
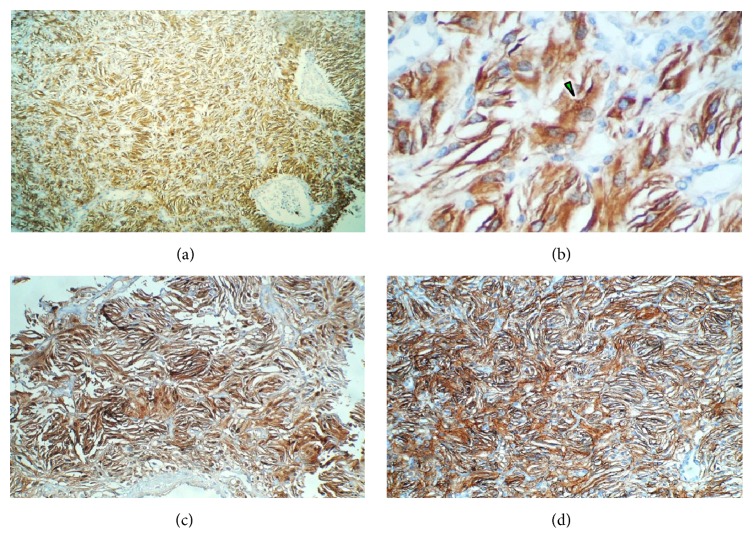
Immunohistochemistry panel. (a) Inhibin A. Ubiquitous expression of this marker in the spindle cell component. (b) Inhibin A. A small hint of lipidization can be identified in one of the giant cells (green arrow). (c) PS100. Confirmative immunostaining of spindle cells. (d) CD57. Corroborative immunolabeling of fusiform cells.

**Figure 4:**
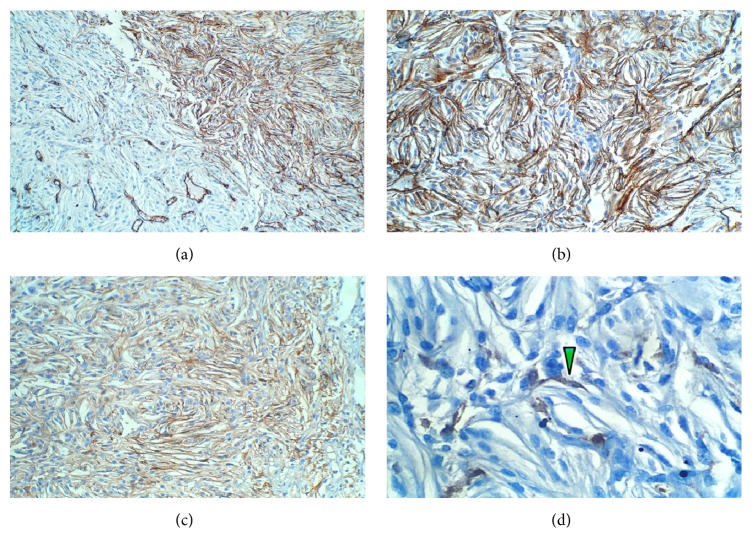
Extended immunohistochemistry panel. (a) CD34. Boundary zone with immunoreactive fusiform cells (right field) as well as nonreactive spindle cells (left field). Positive internal controls—endothelial cells—are identified in the latter. (b) CD34. Closer magnification of the spindle cell component carrying this antigen. (c) CD99. Expression of this marker was also confirmed. (d) FXIIIa. Immunolabeling confined to single cells (green arrow).

In all the samples received, the lesion was constituted by areas of conventional stromal cells admixed with broad regions of slim, short-spindled cells lacking any lipid content and packed in lobules (Figures 2(a)–2(f)). The reticulin pattern was often present around blood vessels; nevertheless, it did not envelop most of the spindled and conventional stromal cells (Figure 2(b)). Interestingly, the last sample also unearthed the presence of a small number of multinucleated, syncytium-looking giant cells scattered through the same fields as the spindled cells (Figures 2(d) and 2(e)). Immunolabeling of both, spindle and giant stromal cells, confirmed the expression of inhibin A, neuron-specific enolase (*not shown*), PS100, and CD57 (Figures 3(a)–3(d)). Intriguingly, local immunostaining of CD34 and CD99 was also identified as well as presence of FXIIIa in isolated cells (Figures 4(a)–4(d)). No reaction was detected towards GFAP, Bcl-2, D2-40, or brachyury; also, ki-67 labeling index was lower than 1% (*not shown*).

## 3. Discussion

Conventional stromal cells in haemangioblastomas are described as round or polygonal in shape either with a lipid-filled microvacuolated cytoplasm or palely eosinophilic and devoid of lipids; nonetheless, a miscellaneous* façade* is the rule [1]. Indeed, spindle glia-like cells are on record [2], but they tend to be long, discohesive, and disorganised when adopting this form; some may even be GFAP positive. Interestingly, the spindle cell morphology seems to be more common among peripheral haemangioblastomas [3], yet they usually are plump or poorly defined in these settings. Regarding the giant cell phenotype, it is also a change kept on registries, though they are just larger versions of vacuolated stromal cells [4]. Multinucleated giant cells, on the other hand, are hardly ever seen. Formerly, Ono et al. [5] reported the case of a cerebellar haemangioblastoma which, unlike ours, showed a distinctly pleomorphic component including multinucleated giant cells and a MIB-1 labeling index of about 8%.

It is currently known that stromal cells in haemangioblastomas harbour markers indigenous to embryonal progenitor cells and have an impending capacity to spawn plurivergent lineages in an appropriate* milieu* [6–8]. In this regard, there seems to be a subset of haemangioblastomas that assume unconventional morpho- and immunophenotypes owing to the influence of multiple fortuitous factors. For instance, Tomono et al. [9] recently documented an unconventional case of haemangioblastoma with rhabdoid features displaying accumulation of intermediate filaments but preserving INI-1 immunostaining. They classified their case as a cellular variant of haemangioblastoma due to the paucity of reticulin fibers among confluent sheets of rhabdoid cells. According to this criterion, our case also belongs to the cellular variant despite having broad areas of spindle shaped cells.

Differential diagnoses in our setting might include spindle cell lipoma as well as fat-forming solitary fibrous tumour; nevertheless, the absence of single-vacuolated, mature adipocytes easily discards them. Likewise, they would be extraaxial tumours in imaging scans. Another interesting option is the so-called “*angioglioma,*” a controversial term that has been indifferently applied to gliomas with degenerative and overflowing vascular changes, arteriovenous malformations with an exuberant gliotic reaction, collision tumours containing both gliomatous and haemangioblastomatous elements, or even the cellular variant of haemangioblastoma itself [10–12]. Bearing in mind the third assumption, one might wonder if our case could possibly represent a collision tumour blending a low-grade astrocytoma with classical haemangioblastoma—as in the recently reported case illustrated by Li et al. [12]. However, we should point to the fact that the spindle cell population described herein was GFAP(−) as well as inhibin A(+), thus confirming their true stromal cell immunophenotype.

As chimaeric as they are, stromal cells in haemangioblastomas may be prone to adopt unexpected morpho- and immunophenotypes that could be related to microenvironmental influences. Thus, rather than uncertain* histogenesis*, it may in fact reflect an ambiguous* histomimesis*—an attempt to recapitulate multiple lines of differentiation. Nevertheless, our case may just simply represent an extensively delipidized form of haemangioblastoma with spindle cell shape-shift. In spite of the latter assumption, CD34 immunoreactivity may be better explained by the former proposal, though we currently do not have the tools to test this hypothesis.

Hence, we communicate a case of haemangioblastoma with unconventional traits emphasising the need to document odd cases in order to better understand this neoplasm.
